# Precision medicine driven by cancer systems biology

**DOI:** 10.1007/s10555-017-9662-4

**Published:** 2017-03-07

**Authors:** Fabian V. Filipp

**Affiliations:** Systems Biology and Cancer Metabolism, Program for Quantitative Systems Biology, University of California Merced, 2500 North Lake Road, Merced, CA 95343 USA

**Keywords:** Cancer systems biology, Melanoma, Subtype, Cancer metabolism, Oncometabolite, Epigenomics, CSD, SCNA, Driver, Big data, Precision medicine, Personalized medicine, Neoantigen, Immunotherapy, Combination therapy, PD1, PDL1, CTLA4, BRAF, MEK, SWI/SNF, ARID1A, ARID2, PRC2, EZH2

## Abstract

Molecular insights from genome and systems biology are influencing how cancer is diagnosed and treated. We critically evaluate big data challenges in precision medicine. The melanoma research community has identified distinct subtypes involving chronic sun-induced damage and the mitogen-activated protein kinase driver pathway. In addition, despite low mutation burden, non-genomic mitogen-activated protein kinase melanoma drivers are found in membrane receptors, metabolism, or epigenetic signaling with the ability to bypass central mitogen-activated protein kinase molecules and activating a similar program of mitogenic effectors. Mutation hotspots, structural modeling, UV signature, and genomic as well as non-genomic mechanisms of disease initiation and progression are taken into consideration to identify resistance mutations and novel drug targets. A comprehensive precision medicine profile of a malignant melanoma patient illustrates future rational drug targeting strategies. Network analysis emphasizes an important role of epigenetic and metabolic master regulators in oncogenesis. Co-occurrence of driver mutations in signaling, metabolic, and epigenetic factors highlights how cumulative alterations of our genomes and epigenomes progressively lead to uncontrolled cell proliferation. Precision insights have the ability to identify independent molecular pathways suitable for drug targeting. Synergistic treatment combinations of orthogonal modalities including immunotherapy, mitogen-activated protein kinase inhibitors, epigenetic inhibitors, and metabolic inhibitors have the potential to overcome immune evasion, side effects, and drug resistance.

## The impact of genomics and systems biology on precision medicine

**Fig. 1:**
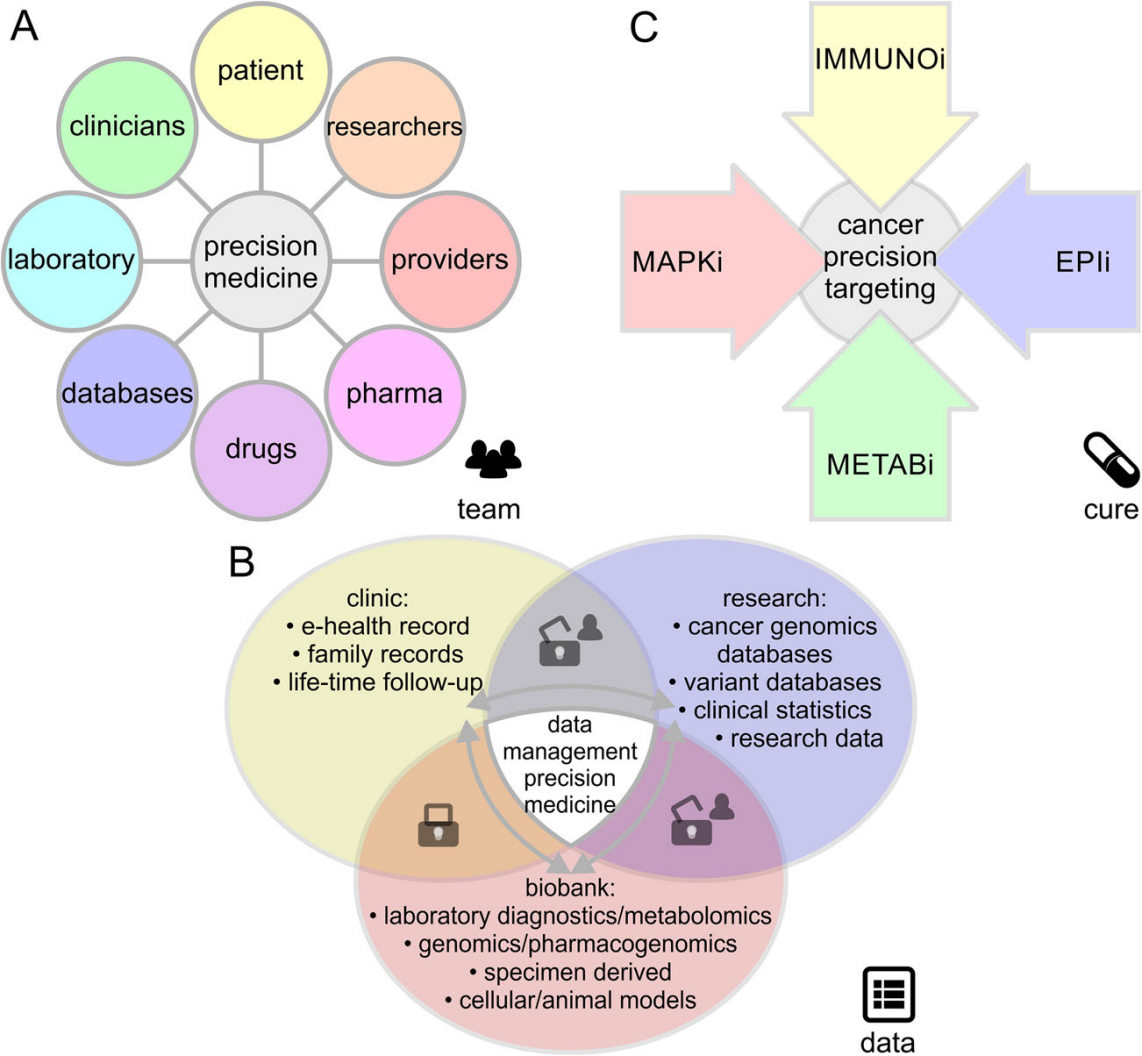
Essential components in precision medicine and its application to cancer. The precision medicine infrastructure relies on a fruitful interplay between **a** a collaborative research team of clinicians and scientists, **b** personalized data allowing for fast and seamless interpretation, and **c** targeted pharmacological strategies. Precision disease management comprises targeted, personalized treatment aligned with the patient’s genotype offering confidence to receive/provide care and hope for cure. In malignant melanoma, rational, orthogonal combinations of immune checkpoint inhibitors (IMMUNOi), epigenetic inhibitors (EPIi), metabolic inhibitors (METABi), or inhibitors of specific signaling pathways such as the mitogen-activated protein kinase inhibitors (MAPKi) will provide best standard of care. Balanced and effective data sharing is based on patient consent, secure data exchange, and synergistic, standardized data formats

The emerging field of precision medicine has formalized our wish for a fast, efficient, and accurate course of action to maximize health benefits (Fig. 1, Box 1). Data-driven science has crossed over into the clinical realm. In an ideal world, precision medicine decisions fit the patient’s needs like a key into a lock. In cancer, each tissue is defined by a distinct molecular signature of genomic and epigenomic changes. Therefore, leveraging genome-wide and systems-wide insights for rational treatment of cancer is the logical, inevitable next step towards data-enhanced disease management and improved survival.

**Box 1:** Definitions of terminology in precision medicine, genome sequencing, and cancer systems biology

Term	Definition
Precision medicine	Medical model tailored to the individual patient. Integrates research knowledge from biomarkers and genomic profiles to accomplish quick, efficient, and accurate medical predictions and decisions.
Big data	Data sets outsize traditional data management and processing. Scale, complexity, and dynamics require new architecture, techniques, algorithms, and analytics to manage it and extract value from it. Big data can be identified by V-type characteristics (see Table 1). Big data analysis can reveal trends, patterns, and correlations based on network interactions.
Genomics	Study of all genes in the human genome. Genomics aims at a full collection of genes and mutations, both inherited and somatic that contribute to the development of an organism, as well as its normal homeostatic function or diseased states. Genome is a portmanteau of the words gene and chromosome.
Epigenomics	Chemical modifications that do not change the DNA sequence but can affect gene activity. Epigenomics refers to capturing epigenetic marks on a genome-wide scale, such as DNA methylation and post-translational modifications of histone chromatin, while epigenetics focuses on regulatory factors and reversible processes affecting gene expression, non-genomic inheritance, and phenotypes that are not the result of variations in DNA sequence.
Systems biology	Science of quantifying, modeling, and visualizing network interactions. Once a model is formulated, systems biology cycles between testable hypotheses and experimental validation.
Genome sequencing	Collection of methods to sequence an entire genome in a single run. The technology involves the capture of fragmented genomic DNA by oligonucleotide probes that collectively cover all exonic or whole genomic sequence regions (abbreviated as WES or WGS, respectively).
Genotyping	The process of determining the genetic makeup of an individual. This can include an entire genome or be targeted to regions associated with a clinical phenotype.
SNP	Variation at a single position in a DNA sequence among individuals or chromosomes. If more than 1% of a population does not carry the same nucleotide at a specific position in the DNA sequence, then this variation can be classified as a single-nucleotide polymorphism, abbreviated SNP and pronounced as snip. SNPs are not just associated with genes or diseases; they can also occur in non-annotated regions of DNA.
SCNA	Somatic copy number alterations, abbreviated SCNAs, are multiplications of deletions of chromosome arms of focal regions that have arisen in a non-germline tissue for example just in a tumor. In contrast, copy number variations, abbreviated CNVs, originate from changes in germline cells and are thus in all cells of the organism.
Master regulator	Rate-limiting step positioned at top of hierarchy. Topologically, master regulators are found at branch points of networks influencing metabolic flux or signaling pathways related to cellular decisions on proliferation, survival, or differentiation.
MAPK pathway	Chain of signaling molecules that communicates a signal from a cell surface receptor into the nucleus of the cell to control a gene expression program including proliferation, mitosis, differentiation, and cell survival. A mitogen-activated protein kinase, abbreviated as MAP kinase or MAPK is part of a kinase family specific to phosphorylation of amino acids serine, threonine, and tyrosine. MAPKs are operated as switches in an amplifying cascade, where phosphorylation of one kinase results in activation of the next kinase. Oncogenic mutations can result in constitutively activated kinases, thereby triggering uncontrolled cell proliferation. Major pathway that is dysregulated in malignant melanoma.
Immunotherapy	Engages certain parts of a person’s immune system to fight diseases such as cancer. This can be accomplished by antibodies, vaccines, or stimulation of the immune system. Immune evasion is a hiding mechanism of cancer cells, which manage to downregulate surface markers that can be recognized by the immune system thereby facilitating unmonitored survival. T-cell immune checkpoint inhibitors silence control elements of the immune system and result in enhanced immune recognition and attack of cancer cells.
Neoantigen	Newly formed epitope that has not been previously recognized by the immune system. New epitopes can arise as a consequence of tumor-specific mutations. Based on such epitopes, the immune system can respond or suppress tumor-reactive T-cell populations, thereby playing a major factor in the activity of immunotherapies such as T-cell checkpoint blockade and adoptive T-cell therapy.
Oncometabolite	Small molecule whose accumulation leads to cancer initiation and progression by metabolic and signaling dysregulation. Specific enantiomers of hydroxy-keto acids or amino acids are accumulated as a result of defective enzymes in cancer and cause genome-wide changes of gene expression by acting as inhibitors of regulatory proteins.

The availability of high-throughput omics data, including deep sequencing of large patient cohorts, has challenged the field of oncology to approach the disease from a holistic systems-based perspective. In the past few years, systems biology has matured as a field and has established rules to design predictive, multiscale models. Such systems biology models enable understanding of biochemical networks, discovery of biomarkers, and patient stratification based on unique genomic and non-genomic (e.g., transcriptomic, epigenomic, proteomic, metabolic) profiles. However, without a mechanism to determine the underlying genetic cause of a set of symptoms, it might not be possible to decide which treatment will be most effective *a priori*. Master regulators present distinct vulnerabilities of networks. Their identification is crucial for systems-based rational drug design as well as for disease management by means of precision medicine. In a cyclic exercise, system biologists derive synergy from complex, multidimensional datasets by hypothesizing, testing, validating, improving, and hypothesizing again to eventually arrive at the understanding that biological networks are stronger than just a plain sum of individual nodes. Cancer genome data has unlocked unprecedented opportunities for systems biology and helped to piece together the puzzle of this complex disease. However, acquisition of cancer genome data is fast-paced and big data challenges will need to be overcome in order to translate genomic insights rapidly into new cancer therapies (Table 1).

**Table 1:** Big data challenges in precision medicine and cancer systems biology

Big data characteristic	Definition	Big data challenges in precision medicine and cancer systems biology
Volume	Data amount	Complementary omics platforms generate terabytes of raw data for each patient to be processed, analyzed, and interpreted.
Velocity	Data in motion	Necessity to acquire, process, share, stream, and compare data; otherwise, important clinical decisions or milestones may be missed.
Variety	Data in different forms	Data in different formats and from different sources covers all levels of biological information from genomics, epigenomics, transcriptomics, proteomics, metabolomics, fluxomics, and phenomics.
Veracity	Data with uncertainty	Sample quality, heterogeneity, efficiency of alignment, data compatibility, incompleteness, ambiguities, latency, deception, and model approximations are factors contributing to uncertainty in data evaluation.

The origin of malignant melanoma—similar to other cancers—is found in cumulative alterations of our genomes and epigenomes progressively leading to uncontrolled cell proliferation. By disrupting signals controlling cell division and migration, such alterations may eventually lead to invasive malignancies able to metastasize and populate distant sites. The molecular signatures of genomic and epigenomic changes will provide insight as to why melanoma stands out as such a serious and aggressive malignancy. Furthermore, this knowledge will impact our perspective on how to approach melanoma prevention, diagnosis, and therapy. We will provide examples of how systems biology- and genome biology-driven approaches to cancer accelerate our ability to understand and respond to malignant melanoma.

## Melanoma genomics and big data challenges in precision medicine

**Fig. 2:**
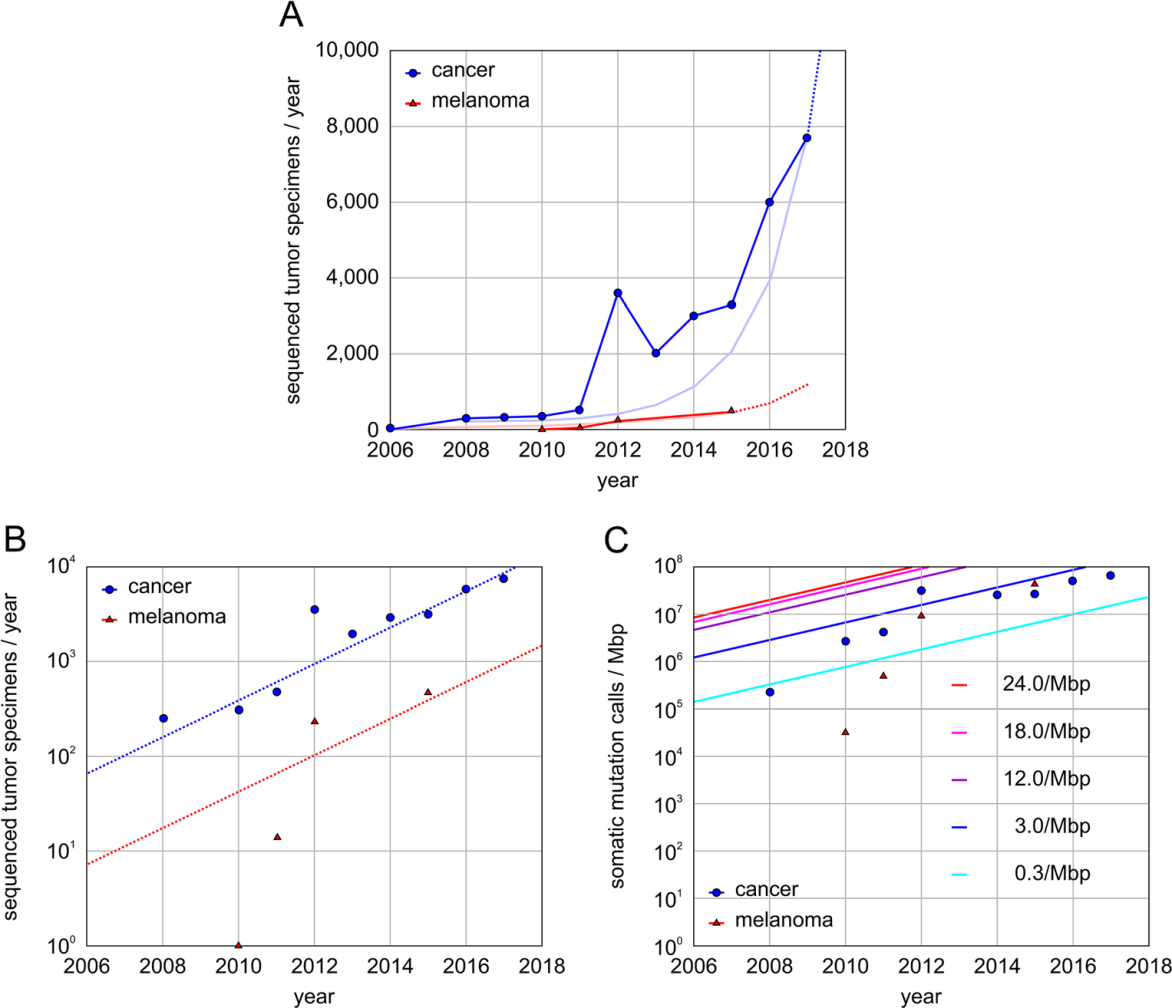
Rate and challenges of cancer genomics studies with sequenced tumors. **a** Sequenced and publically available cancer genomics studies per year. The data contains cancer genomics studies with a total of more than 20,000 whole exome sequenced or whole genome sequenced tumor specimens. Exponential *trend lines* are based on current approximate slope. Extrapolated data indicated by *dashed line*. **b** Exponential increase and trend lines of whole genome and whole exome sequenced specimens per year for melanoma and studies across all tissues (Pan-cancer). **c** Melanoma specimens with high mutational burden carry numerous somatic alterations and require somatic mutation calls at high frequency. Depending on cancer tissue, cohort size, and mutational burden, the dynamic range of somatic mutation calls can span several orders of magnitude. Average *trend lines are* shown for different cancers tissues with low (*cyan*), medium (*blue*), and high (*purple*) mutational burden. Despite the fact that the currently available melanoma cohort covers less than a tenth of all cancer tissues, the data demands of somatic mutation calls in melanoma genomics are equally challenging to that of other current cancer genomics studies combined

The field of melanoma genomics has experienced an exponential rise over the past decade and has rapidly become a paradigm for providing insight into melanoma driver genes, cancer signaling, tumor immunology, and drug targets (Fig. 2). Next-generation sequencing technology has made it possible to capture alterations by different platforms and biological levels such as RNA-Seq transcriptome analysis [1], sequencing of microRNAs (miRNA-Seq) [2, 3], whole exome sequencing (WES) [4, 5], whole genome sequencing (WGS) [6–8], and chromatin immunoprecipitation with next-generation sequencing (ChIP-Seq) [9]. In addition, high-throughput single-nucleotide polymorphism (SNP) arrays [10, 11] have accelerated progress in the field of somatic copy number alterations (SCNAs) [12]. SCNAs can be captured either by low-coverage WGS or SNP arrays [13].

The range of observed mutation rates within and across tumor types spans more than five orders of magnitude (between 0.03 and 7000/Mbp) [14]. Melanoma was one of the earliest tumor types sequenced, contributing to the forefront of cancer genomics. The first comprehensive catalog of somatic events was established by WGS on the metastatic COLO-829 cell line derived from a malignant melanoma patient [15]. Cancer genomics experienced a peak in 2012 (Fig. 2a), when many studies from The Cancer Genome Atlas (TCGA) matured, publically funded by the National Cancer Institute [16–18]. In a collaborative effort, thousands of tumor biopsies were deposited in bio-specimen repositories, sequenced, processed at individual data levels, merged, and analyzed. Similarly, the number of melanoma genomes sequenced broke the hundreds in the same year [19–22]. However, the ability of melanoma genomics to keep up with the pace and trends in other cancer genomics fields was temporarily hampered by the disproportionate data demands and frequent somatic mutation calls (Fig. 2b, Box 1). In fact, the TCGA skin cutaneous melanoma (SKCM) studies of 2015 required more somatic mutation calls (average rate of 18/Mbp; many individual specimens with rates above 1000/Mbp) than all other TCGA genomics studies combined [13, 23] (Fig. 2c). The bioinformatics tool Introns Vs Exons (InVEx) [20], designed to identify driver genes by taking the specific background mutation rate per patient and per gene into consideration, is currently the gold standard in melanoma genomics. Genome-wide capture of significant somatic events in melanoma is computationally intensive. Currently, more than 700 melanoma specimens with WES are available, comprising about 4% of the overall cancer genomics efforts (Fig. 2a). Given the current 1.6-fold increase in data production and exomes sequenced per year, it is expected that soon cancer studies across all tissues can accomplish 10,000s and melanoma studies 1000s of specimens/year. Sharable data formats like oncotator will facilitate future meta-studies of public and private melanoma genomics efforts [24]. According to the saturation analysis of cancer drivers, it will take more than 5000 specimens to reach more than 99% power for a comprehensive catalog of cancer drivers with intermediate mutation frequencies of 2–5% in melanoma [14, 25]. Despite that the search for melanoma driver genes is not saturated, the cancer research community has already established a consistent set of high-frequency driver genes.

Today’s big biomedical data contains large, complex data structures. In addition, comprehensive coverage requires different data levels, formats, and sources. V-type challenges including volume, velocity, variety, and veracity (Table 1) need to be overcome in order to link, match, cleanse, and transform data across systems. Big data challenges are already apparent at the molecular level for an individual patient when looking at a precision medicine profile. Furthermore, interpretation of precision medicine data for large patient cohorts requires connection and correlation, hierarchies, and multilevel linkages. Systems biology has the unique ability to connect nucleotide or gene-based next-generation sequencing data with other functional levels such as protein structures or metabolite fragments acquired by high-throughput spectroscopy or spectrometry methods. Tomorrow’s big data in precision medicine requires savvy solutions to capture, curate, store, share, maintain, update, query, and visualize data while maintaining agreed privacy standards.

**Fig. 3:**
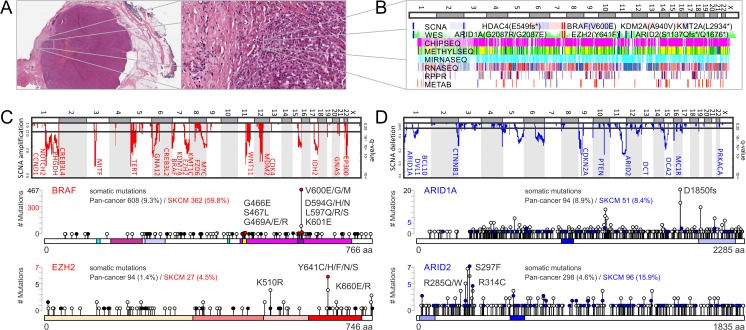
Precision medicine profile and rational drug targeting of malignant melanoma. **a** Whole-slide tumor tissue image of malignant melanoma shows tumor microenvironment and its impact on metabolically and mitotically active melanoma cells. **b** Multiomics data of tumor specimen is matched with high-throughput functional genomics data of cellular cultures. The personalized precision medicine *chart* shows deregulation of important signaling and epigenetic molecules across matched data tracks of genomic, epigenomic, transcriptomic, proteomics, and metabolomics platforms. **c** Comparison of patient data with somatic copy number alterations (SCNAs) of melanoma cohort identifies significant amplifications (*red*) and **d** deletions (*blue*). Detected somatic alterations of BRAF and EZH2 coincide, fall into mutational hotspots, and result in gain-of-function oncogenes. The somatic landscape of ARID1A and ARID2 is characterized by somatic non-sense and missense mutations, which result in loss of function of a tumor suppressor complex involved in chromatin remodeling. **e** Rewiring of metabolism and metabolic signaling affects melanogenesis and tunes central carbon metabolism to support evasion, proliferation, and survival of malignant melanoma. **f** Oncometabolites impact the epigenetic machinery by blocking or supplying carbons for histone modifiers. Epigenetic master regulators in cancer control transcriptional activation of other oncogenes or repression of tumor suppressors. **g** Personalized medicine strategies to overcome treatment resistance of malignant melanoma patients. **h** Patient profiles of The Cancer Genome Altas (TCGA) reveal co-occurrence of BRAF and EZH2 hyperactivity making combination therapy of mitogen-activated kinase inhibitors (MAPKi) and epigenetic inhibitors (EPIi) viable. As alternative option if immunotherapy (IMMUNOi) fails due to immune evasion and suppression of immune receptors, combination therapy of IMMUNOi and metabolic inhibitors (METABi) is sensible

A successful approach to melanoma precision medicine will take an integrated, seamless team effort of clinicians, researchers, and providers (Fig. 1a). While genetic insights are quickly adopted into the clinical decision-making process, the infrastructure for genomics and precision medicine yet has to be established. Deep sequencing efforts of melanoma specimens are not well-integrated into the clinical routine, and cohort-based genomics tools like GISTIC [26], MutSigCV [27], or InVEx [20] are rather built for discovery research [13] rather than day-to-day diagnostics. Patient consented data sharing is necessary to securely exchange data between clinical caretakers, biobanks, omics data acquisition platforms, cloud-based processing, and genomics researchers. Not surprisingly, conservative models of privacy were challenged long before the advent of precision medicine by direct-to-consumer genetic tests and population genome research projects [28–31]. Instead of relying on generalizable knowledge, tomorrow’s repositories are moving towards fully consented models motivated by a main benefit of being able to associate longitudinal health records [32–34] (Fig. 1). A first step, the rebranding of personalized medicine to precision medicine, has been successfully undertaken and resulted in broad public approval in contrast to the early rhetoric of personalized medicine [35]. Genomic and epigenomic testing will be a useful tool to increase therapeutic success rates. Genetic markers hold a well-defined probabilistic value, but when it comes down to it, clinical implementation is important to address ethical boundaries including stigmata of genetic determinism. Reexamination and an update of ethical standards to catch up with rapidly evolving innovations in precision medicine, forensics, and population genomics seem inevitable.

Early on, identification of activating point mutations in the mitogen-activated protein kinase (MAPK) pathway in B-Raf proto-oncogene (BRAF, serine/threonine kinase, Gene ID: 673) [36, 37] and neuroblastoma RAS viral oncogene homolog (NRAS, Gene ID: 4893) made it possible to match genotyped kinase mutations with precision medicine approaches based on specific kinase inhibitors [38, 39]. Although BRAF-activated melanomas initially have been the most successful melanoma subset due to targeted therapies, they will ultimately develop resistance and there is rapid progress in complementary approaches especially ones taking advantage of the immune system to fight cancer cells. In principle, melanoma can be targeted from complementary angles, including immune-checkpoint inhibitors (IMMUNOi) and/or targeted therapy with inhibitors of specific signaling pathways such as the mitogen-activated protein kinase inhibitors (MAPKi), epigenetic inhibitors (EPIi), or metabolic inhibitors (METABi) (Fig. 1c).

The survival of advanced metastatic melanoma patients has dramatically improved due to second-generation clinical trials of melanoma drugs involving regimes with targeted therapy or immune-checkpoint antibodies. For both groups, combinations proved to be superior to single-agent regimens: Inhibition of BRAF in combination with mitogen-activated protein kinase kinase 7 (MAP2K7, MEK, Gene ID: 5609) improved survival compared to single MAPKi [40–43]; blockade of combined immune checkpoints by humanized antibodies improved survival compared to single IMMUNOi of cytotoxic T-lymphocyte associated protein 4 (CTLA4, Gene ID: 1493) with programmed cell death 1 (PDCD1, PD1, CD279, Gene ID: 5133) or CD274 molecule (CD274, PDL1, PDCD1L1, Gene ID: 29126) [44–48] (Table 2).

Specific small-molecule agents and respective trials include BRAF MAPKi: vemurafenib (PLX4032), dabrafenib (GSK2118436), and encorafenib (LGX818) [38, 49–51]; MAP2K7 (MEK) MAPKi: trametinib (GSK-1120212) and cobimetinib (GDC-0973) [39]; combination of BRAF and MAP2K7 MAPKi [40–43, 52–54]; CTLA4 IMMUNOi: ipilimumab (MDX-010) and tremelimumab (CP-675206), [55–59]; PDCD1 (PD1) IMMUNOi: nivolumab (MDX-1106, Opdivo) and pembrolizumab (MK-3475, Keytruda) [60]; CD274 (PDL1) IMMUNOi: atezolizumab (Tecentriq); and combinations of PDCD1 and CTLA4 IMMUNOi [44–46, 61].

Of single-agent therapies, conventional chemotherapy or CTLA4 blockade showed poorest outcomes [62]. However, pilot clinical data directly evaluating efficacy of combinations of MAPKi and IMMUNOi is promising. Despite frequent relapse and considerable side effects, targeted therapies show a remarkable ability to suppress tumor growth and lead in addition to a better functioning immune system [63]. This may assist durable response of immunotherapy in future combination trials involving MAPKi and IMMUNOi. It can be foreseen that third-generation trials integrating precision medicine insights, new combinations or schedules, and the less explored classes of EPIi and METABi will further improve already efficacious regimens. In particular, cancer systems biology offers insight into immunosuppressive effects of genomic, epigenomic, and metabolic drivers.

**Table 2:** Clinical trials with single-agent regimens or combinations of melanoma MAPKi and IMMUNOi drugs

Inhibitor	Gene target	Trials	Drug name	Compound	PubChem	Trade
MAPKi	BRAF	[38, 49–51]	Vemurafenib	PLX4032	CID: 42611257	Zelboraf
			Dabrafenib	GSK2118436	CID: 44462760	
			Encorafenib	LGX818	CID: 50922675	
MAPKi	MAP2K7 (MEK)	[39]	Trametinib	GSK-1120212	CID: 11707110	
			Cobimetinib	GDC-0973	CID: 16222096	
MAPKi	Combination of BRAF and MEK inhibition	[40–43, 52–54]				
IMMUNOi	CTLA4	[55–59]	Ipilimumab	MDX-010	SID: 131273201	
			Tremelimumab	CP-675206	SID: 47208308	
IMMUNOi	PDCD1 (PD1)	[60]	Nivolumab	MDX-1106	SID: 135341610	Opdivo
			Pembrolizumab	MK-3475	SID: 187051801	Keytruda
IMMUNOi	CD274 (PDL1)		Atezolizumab	1380723-44-3	SID: 312642102	Tecentriq
IMMUNOi	Combination of PDCD1 and CTLA4 blockage	[44–46, 61]				

Current clinical trials utilize small-molecule mitogen-activated protein kinase inhibitors (MAPKi) or humanized antibodies as immune checkpoint inhibitors (IMMUNOi). Molecules are referenced based on their unique PubChem chemical identifier CID or substance identifier SID

Because relapse, disease progression, or high rates of autoimmune toxicity still need to be overcome, precision medicine in cancer offers hope for better long-term benefits and reduced side effects. While the clinical relevance of immune cells in the control of human cancers is well established and genome sequencing captures the somatic landscape, neoantigens that arise as a consequence of tumor-specific mutations are a major factor in the activity of immunotherapies [64–73]. Further, neoantigens present themselves as targets for personalized vaccines and cell therapies that target patient-specific cancer mutations [74–79]. The identification of neoantigens can be assisted by structure-based prediction or modeling approaches [80]. Predictions of drug responses in melanoma will eventually improve by looking into gene expression signatures associated with immune sensitivity or resistance [81].

## Precision medicine profile of malignant melanoma patient with BRAF, EZH2, and ARID signature

**Fig. 4:**
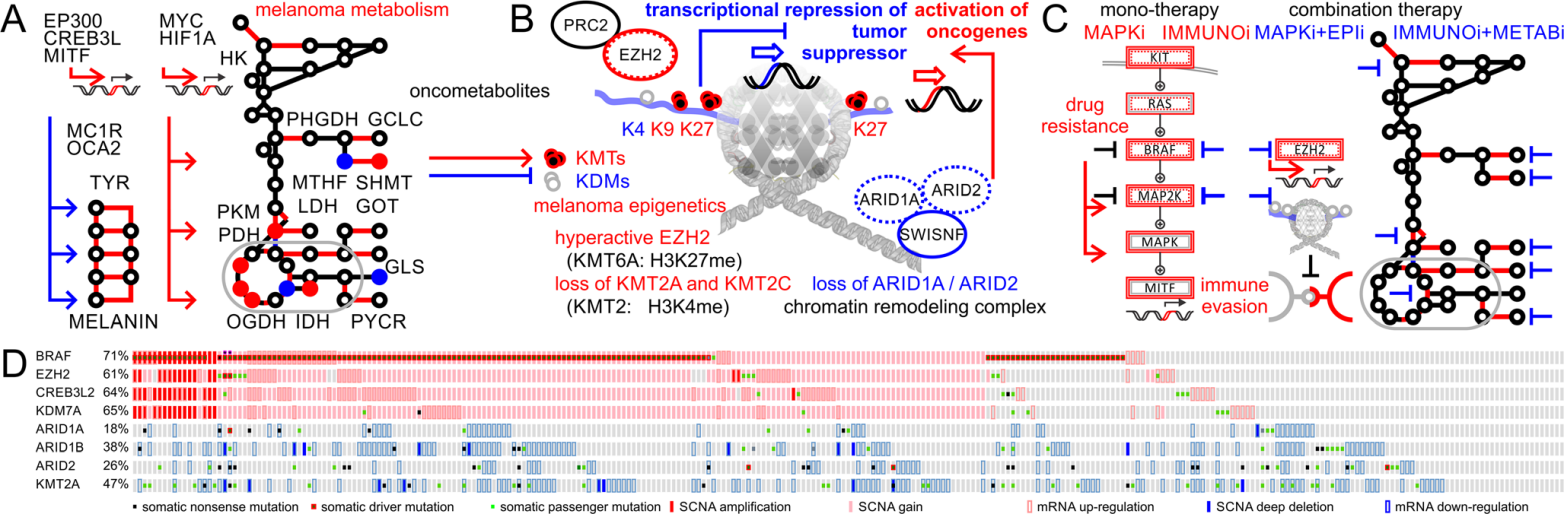
Precision medicine profile and rational drug targeting of malignant melanoma. **a** Rewiring of metabolism and metabolic signaling affects melanogenesis and tunes central carbon metabolism to support evasion, proliferation, and survival of malignant melanoma. b Oncometabolites impact the epigenetic machinery by blocking or supplying carbons for histone modifiers. Epigenetic master regulators in cancer control transcriptional activation of other oncogenes or repression of tumor suppressors. **c** Personalized medicine strategies to overcome treatment resistance of malignant melanoma patients. **d** Patient profiles of The Cancer Genome Atlas (TCGA) reveal co-occurrence of BRAF and EZH2 hyperactivity making combination therapy of mitogen-activated kinase inhibitors (MAPKi) and epigenetic inhibitors (EPIi) viable. As alternative option, if immunotherapy (IMMUNOi) fails due to immune evasion and suppression of immune receptors, combination therapy of IMMUNOi and metabolic inhibitors (METABi) is sensible

Genotyping has already replaced phenotyping in diagnosing selected Mendelian disorders, neurologic syndromes, and in prenatal or neonatal care [82, 83]. In cancer care, phenotype-driven gene target definition is typical and clinical genome-wide sequencing data interpretation is still accompanied by a large-scale research effort [84]. Here, we illustrate a complete precision medicine profile of a malignant melanoma patient [85] (Figs. 3 and 4). Cancer tissue image collections from a variety of imaging modalities, including whole-slide tissue images, computed tomography, magnetic resonance, X-ray, or positron-emission tomography, allow pathologists to make novel genotype-phenotype associations enabled by correlative big-data studies [86, 87]. Automated computational analysis and morphometry of whole-slide images has the unique ability to investigate tumor microenvironmental drivers of disease progression—an important aspect that is not covered by any other platform (Fig. 3a). Genome-wide matched omics data enables correlations at the chromosome, gene, and nucleotide levels (Fig. 3b). SCNA, WES, DNA methylation data, RNA-Seq, miRNA-Seq, reverse phase protein arrays (RPPA), and metabolomics data cover major levels in the flow of biological information from DNA to distinct subcellular chemical environments. In addition, epigenomics and functional genomics data obtained from cellular cultures of tumor specimens can be added to obtain a comprehensive precision medicine chart (Fig. 3b). The landscape of somatic alterations in malignant melanoma shows significant focal- and arm-level amplifications and clefts (Fig. 3c, d). SCNAs by SNP and low-coverage WGS detected genomic deregulation of many cancer drivers known to play important roles in melanocyte development and tissue homeostasis [13]. Hotspots of somatic events of individual patient data can be compared to cancer genome databases [88]. Mutation pattern in combination with three-dimensional protein structures reveals the mutation distribution of specific functional regions and can highlight hotspots or oncogenic drivers [89–92]. The study on efficient detection of genomic drivers in malignant melanoma identified co-occurrence of a mutational and copy number hotspot on chromosome 7 including the known BRAF locus [13]. Interestingly, the activating somatic BRAF(V600E) mutation is accompanied by somatic mutation and/or somatic copy number amplification of prominent epigenetic regulators such as enhancer of zeste 2 polycomb repressive complex 2 subunit (EZH2, KMT6A, Gene ID: 2146), lysine demethylase 7A (KDM7A, JHDM1D, Gene ID: 80853), and euchromatic histone lysine methyltransferase 2 (EHMT2, KMT1C, Gene ID: 10919) [13, 85]. The detailed analysis of the mutational landscape of BRAF revealed missense mutations in the ATP-binding site of G466, S467, and G489 as well as in the activator loop of D594, L597, and K601 in the proximity of residue V600 [13]. EZH2 contains the catalytic histone lysine methyl transferase subunit of the polycomb repressive complex 2 (PRC2), which plays a role in maintenance of the stem cell-like state of cancer cells and in the progression of malignant melanoma [93]. Hyperactivation of EZH2 by somatic mutations or copy number gain occurs in 20% of melanoma patients and negatively impacts overall survival [85].

## Tumor suppressors and chromatin remodeling complexes carry protective function but impede simple genotyping

Non-coding, non-sense mutations are of particular importance in tumor suppressors, since such factors carry gatekeeping and protective function in healthy tissue. In melanoma, highest ranking tumor suppressor genes are tumor protein p53 (TP53, Gene ID: 7157), neurofibromin 1 (NF1, Gene ID: 4763), AT-rich interaction domain 2 (ARID2, BAF200, Gene ID: 196528), AT-rich interaction domain 1A (ARID1A, BAF250, Gene ID: 8289), cyclin-dependent kinase inhibitor 2A (CDKN2A, P14ARF/P16INK4A, Gene ID: 1029), and phosphatase and tensin homolog (PTEN, Gene ID: 5728). In contrast to oncogenic gain-of-function events, deleterious loss-of-function events of tumor suppressors are usually homozygous but diverse in their molecular profile. The ATP-fueled chromatin remodeling complex switch/sucrose non-fermentable (SWI/SNF) [94, 95] around ARID1A and ARID2 has the ability to make chromatin accessible for gene expression and for repair enzymes counteracting UV-induced DNA damage. Similar to PRC2, the SWI/SNF complex is essential for the self-renewal in melanocyte development and in pluripotent stem cells. A diverse array of somatic copy number deletions and non-sense mutations of epigenetic regulators ARID1A and ARID2 lead to dysfunctional chromatin remodeling (Fig. 3d). Such unprecedented complexity of the mutational landscape of cancer drivers puts simple targeted, single-nucleotide variation-based genotyping assays at a disadvantage. In contrast, genome sequencing has the ability to capture both breadth and depth of somatic alterations in cancer. However, calling of SCNAs and somatic mutations for each patient is just a fraction of the precision medicine workflow. Only in few rare cases of well-characterized genomic alterations it is possible to reliably predict implications at the messenger, protein, and metabolite levels. For effective precision medicine treatment, systems biology interpretation, comparison to human mutation, genomics, and variant databases [31, 96–98], functional connection between different omics platforms, and prediction for drug response are equally important [99] (Figs. 1b and 4a–d).

## Oncometabolites connect melanoma metabolism and epigenetics

No field other than melanoma has better accounted for connections between genetic causation, disease phenotype, and physical presentation: genomic studies have the ability to identify links between pigmentation of skin, hair, and eyes, tissue development, melanogenesis, pigment metabolism, mitogenic signaling pathways, ethnicity, gender, age, susceptibility loci, disease risk, initiation, and melanoma progression (Fig. 4a). Profiling studies of melanoma utilizing metabolomics and fluxomics revealed a distinct decoupling of glycolysis and glutamine-fueled TCA cycle in support of hypoxic survival [100–102]. While metabolic rewiring supported by specific isoforms of metabolic enzymes including hexokinase, phosphoglycerate dehydrogenase, serine methyl transferase, glycine cleavage complex, pyruvate kinase, pyruvate dehydrogenase, oxoglutarate dehydrogenase, isocitrate dehydrogenase, pyrroline-5-carboxylate reductase, glutamic oxaloacetic transaminase, and glutaminase facilitate production of biosynthetic precursors, metabolites can also have functions in signaling and epigenetics [99, 103, 104] (Fig. 4a, b). Overabundance or limitation of onco-metabolites such as amino- and beta-ketoacids [105–107] are direct suppliers and controllers of histone methyl modifiers [108]. Mutant isocitrate dehydrogenase (NADP(+)) 1 (IDH1, Gene ID: 3417) will produce 2-hydroxyglutarate (2HG, (*R*)-hydroxyglutarate, PubChem CID: 439391) and modulate histone lysine demethylases (KDMs) known to be central epigenetic regulators. In contrast, histone lysine methyltransferases (KMTs) like EZH2 rely on a steady supply of S-adenosyl methionine (SAM, PubChem CID: 9865604) utilized for histone tri-methylation leading to repression of tumor suppressors [85, 109]. The emphasis of EZH2 in melanoma is enhanced by specific loss of other KTMs [108].

## Rational combination of orthogonally targeted therapies

The fraction of newly discovered epigenetic players in melanoma genomics is striking and indicates environmental implications beyond UV-induced carcinogenic photoproducts [110]. In contrast to genomics, the emerging field of epigenomics represents only about 2% of the genome-scale studies but grows at a much higher rate [111]. The co-occurrence of BRAF and EZH2 hyperactivation [13, 85] as illustrated in the precision medicine case in melanoma presented above opens new possibilities for targeted treatment. Mono-therapy with MAPKi (either against BRAF or MAP2K7) is known to lead to drug resistance and disease progression. Combination therapies of different MAPKi molecules are effective but have significant side effects. A precision treatment that takes advantage of the independence of signaling and epigenetic pathways could combine MAPKi and EPIi (Fig. 4a). Elevated activity of EZH2 in cancer can be targeted by small-molecule drugs (e.g., GSK126, PubChem CID 68210102) [112] and is associated with sensitivity to apoptotic cell death [109]. Transcriptomic profiling showed that several well-known tumor suppressors such as BCL2 family apoptosis regulator (BOK, BCL2L9L, Gene ID: 666), N-myc downstream regulated 1 (NDRG1, Gene ID: 10397), and Ras association domain family member 5 (RASSF5, Gene ID: 83593) are downregulated in cells with abnormalities in EZH2, which can be reversed by inhibitor treatment [85]. An *in vitro* EZH2 drug targeting assay made it possible to validate genes associated with immune recognition, cytokine, chemokine, and major histocompatibility complex class II antigen presentation [85, 113]. Identified target genes by combination of transcriptomic and ChIP-Seq assessment revealed genes associated with tumor suppression, apoptosis, cell differentiation, cell cycle inhibition, and repression of metastases as well as antigen processing and presentation pathways. EZH2 controls cross-talk with natural killer cells suggesting an immunosuppressive effect of EZH2 involving both innate and adaptive immunity [114]. Cell culture and animal models with EZH2 inhibitors show significantly decreased spheroid growths, cell cycle arrest, apoptosis, and tumor regression [85, 109, 114]. EZH2 enhances immune evasion and tumor proliferation by repressing a defined gene expression program of immune receptor and apoptotic signaling. This raises the question, whether EPIi or METABi with patients of rewired epigenetics and tumor metabolism have the ability to support the efficacy of IMMUNOi. Captivatingly, a metabolic enzyme, lactate dehydrogenase, central to the Warburg effect in cancer is a negative prognosticator for IMMUNOi response and predictive biomarker for overall survival of patients with malignant melanoma [115, 116]. Taken together, patients with somatic activation of EZH2 may benefit from treatment combinations that include inhibitors of EZH2 [85] (Fig. 4c). Given a more than 60% frequency of genomic and non-genomic somatic activation of EZH2 in melanoma patients, this case of precision medicine might be more generalizable and synergy between epigenetic and immune checkpoint inhibition has a strong potential for a broader treatment regimen.

## The ideal personalized strategy is specific but has wide coverage

A major concern of precision medicine approaches is the generalizability of identified somatic events and isolated case studies. Tumor heterogeneity and high background mutation rate in cancers like melanomas further impede one-dimensional disease management that can be expected to work for all patients. Comparisons to genomics databases like TCGA support the significance of the observed co-occurring signature of BRAF with epigenetic regulators (Fig. 4d) [117, 118]. EZH2, cAMP-responsive element binding protein 3 like 2 (CREB3L2, Gene ID: 64764), and KDM7A are not only significantly co-amplified due to their genomic proximity (*p* value below 0.001); their mutational signature shows co-occurrence as well [13, 85]. In addition, important epigenetic regulators with tumor suppressor function are lost in melanoma at the level of somatic copy number deletions or inactivating somatic mutations. Taken together, the observed precision medicine signature representative for any patient with rewired MAPK and epigenetic regulators is likely to be relevant to the majority of the melanoma cohort (229 of 287), taking all genomic, transcriptomic, and proteomic data into consideration (Fig. 4d).

## Melanoma subtypes and predominance of MAPK-related signaling

**Fig. 5:**
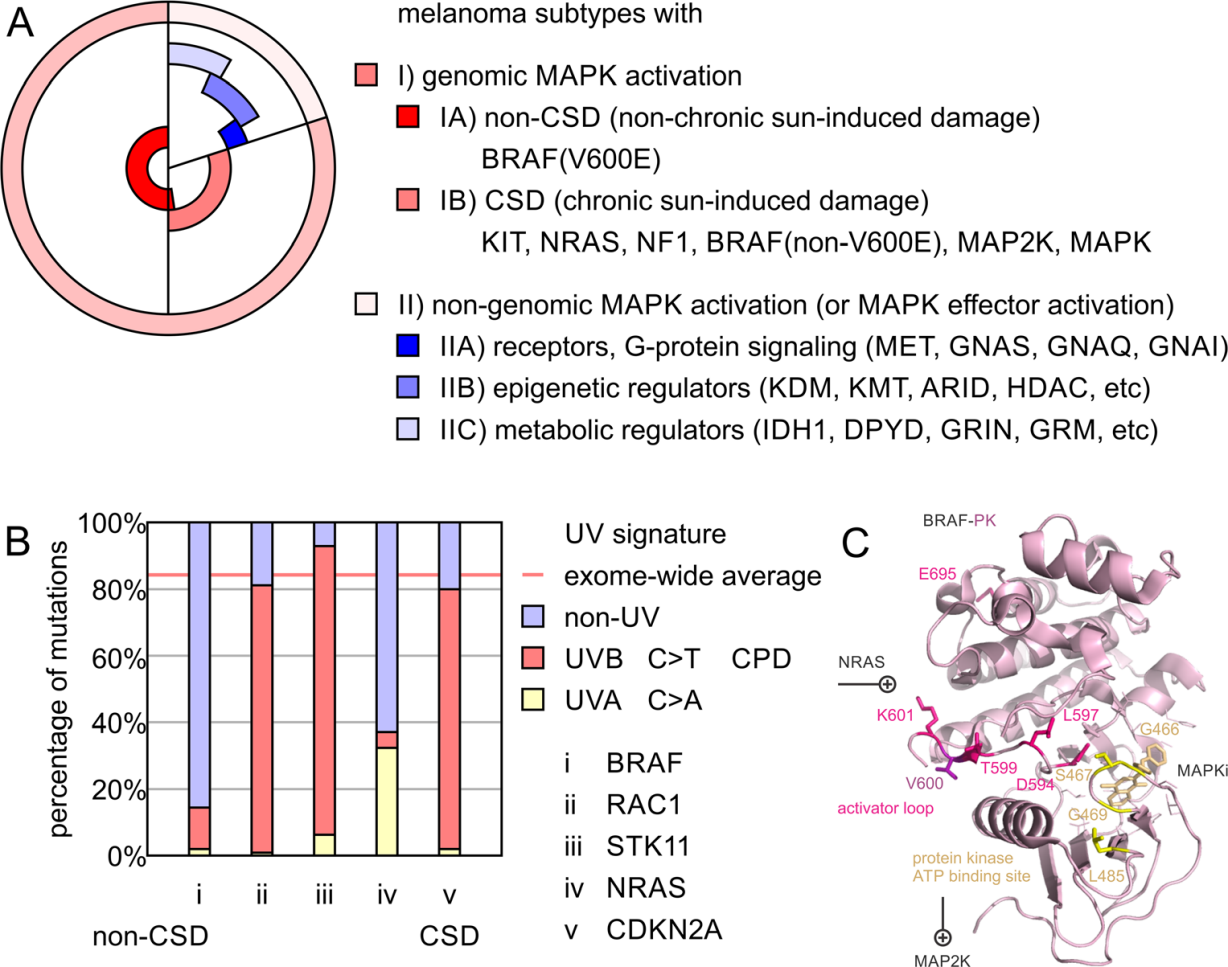
Melanoma subtypes and UV signature of mutagenesis in melanoma drivers. **a** Melanoma subtypes are divided by (I) genomic and (II) non-genomic activation of the mitogen-activated protein kinase (MAPK) pathway. Melanoma with genomic MAPK activation contain (IA) non-chronic sun-induced damage (non-CSD) melanomas with BRAF(V600E) mutation (50%) and (IB) chronic sun-induced damage (CSD) melanomas with mutations of central genes of the MAPK pathway including KIT, NRAS, NF1, BRAF(non-V600E), MAP2K, or MAPK (∼30%). Both non-CSD and CSD share subtype (I) and have genomic activation of the MAPK pathway in common (∼80%). Any melanoma without genomic activation of MAPK elements is defined as subtype (II) (∼20%). Subtype (II) melanomas with low mutational burden are enriched in driver genes of (IIA) membrane receptors and/or G protein signaling, (IIB) epigenetic regulators, and/or (IIC) metabolic regulators. Subtype (II) frequently share effector activation of MAPK signaling by non-genomic mechanisms. **b** UV signature of nucleotide transitions in melanoma is determined by exome-wide genome sequencing. Percentage of driver mutations caused by UVA (C>A) or UVB (C>T) are plotted vs the exome-wide sample average percentage. Example genes **a**–**e** provide representative examples of melanoma drivers: *i* BRAF, *ii* RAC1, *iii* STK11, *iv* NRAS, and *v* CDKN2A. The non-CSD driver gene BRAF contains a high fraction of non-UV mutations deviating from exome-wide sample average; CSD drivers contain nucleotide transversions by cyclobutane pyrimidine dimers (CPDs) responsible for the initiation of the predominant C>T UV melanoma signature mutations. Tumor suppressor genes with high mutational burden and co-occurrence with CSD genes can closely reflect the exome-wide sample average of UV-related base transitions. Oncogenic drivers are subject to molecular evolution selecting for specific base transitions, which can cause deviation of the exome-wide sample average. Structural representation of non-CSD and CSD mutations of melanoma driver BRAF. Residue V600 in the center of the activator loop of BRAF is highlighted in *purple*. The comprehensive somatic molecular landscape of BRAF in melanoma is illustrated in Figs. 3 and 4. CSD hotspots include the activator loop in *pink* and the ATP-binding site in *yellow*

Genomic insights identified distinct molecular signatures of malignant melanoma with important consequences for patient stratification (Fig. 5). Mutual exclusivity of oncogenic events in the MAPK pathway covers about three fourths of the malignant melanoma patient cohort [13, 23, 25]. On the one hand, melanomas with chronic sun-induced damage (CSD) harbor mutations in KIT proto-oncogene receptor tyrosine kinase (KIT, Gene ID: 3815), NF1, NRAS, and BRAF but typically not BRAF(V600E), the specific BRAF mutation that commonly arises in atypical melanocytic neoplasms [119, 120]. On the other hand, non-CSD melanomas show a predominance of BRAF(V600E) mutations arising from nevi [121]. Both types have core activation of the MAPK pathway in common, covering at least 80% of the melanoma cohort (Fig. 5a). Since BRAF(V600E) activation requires a T>A transversion at nucleotide 1799, BRAF non-CSD mutations do not resemble nucleotide transitions typically found in sun-induced melanoma damage arising from cyclobutane pyrimidine dimers (CPDs) (Fig. 5b). CPDs are DNA photoproducts of joined adjacent pyrimidines and responsible for the initiation of the predominant C>T UV melanoma signature mutations [122]. UV-induced reactive oxygen species have the ability to oxidize melanin to create melanin carbonyls. This explains why carcinogenic CPDs can be generated even after UV exposure of the skin ends [123]. Both oxidative DNA damage and UV-induced mutation signatures have an overlapping footprint related to pyrimidine heterocycle chemistry. Future chemoprevention efforts of malignant melanoma may take advantage of such prevalent molecular features connecting mutation signature, pigmentation genetics, and cellular environment [124]. Similar to BRAF, melanoma driver genes like ras-related C3 botulinum toxin substrate 1 (RAC1, rho family, small GTP binding protein Rac1, Gene ID: 5879), serine/threonine kinase 11 (STK11, LKB1, Gene ID: 6794), or NRAS have such a distorted distribution of nucleotide transitions, since activation nucleotide transversions can favor or disfavor the UV melanoma signature. A CSD UV signature is typically found in melanoma tumor suppressors such as CDKN2A resembling the exome average.

## Activation of the MAPK pathway by genomic and non-genomic mechanisms

Activation of BRAF alone by somatic mutation, amplification, transcriptional upregulation, or post-translational modification is present in more than 80% of the patients. In addition to BRAF(V600E) non-silent, polar replacements in the activator loop (p.D594G/H/N, p.G596D/R/S p. L597Q/R/S, p.T599TT, p.K601E/N/T), the glycine-rich ATP binding site (p.G466A/E/V, p.S467L, p.G469A/E/L/R/S/V), or the protein interaction surfaces of BRAF activate MAPK signaling (Fig. 5c). In the case of genomic MAPK activation (subtype I), the BRAF status as well as signature UV mutations provide insight into the molecular origin of melanoma. Knowledge of subtype IA vs IB is of importance for the choice of the drug regime, where BRAF(V600E) mutations were the prime target scaffold for small-molecule design [38, 125, 126]. Both CSD and non-CSD subtypes share genomic activation of core elements of the MAPK pathway. The remaining non-genomic MAPK subtype (also referred to as BRAF/NRAS/NF1 wild-type subtype) predominates in younger individuals with overall lower mutation burden and heterogeneous origin. However, at low frequency, non-genomic MAPK melanoma drivers are found in pathways of G protein-coupled receptors, metabolism, or epigenetic signaling (Fig. 5a). Overexpression of receptor tyrosine kinases platelet-derived growth factor receptor beta (PDGFRB, PDGFR, Gene ID: 5159) or MET proto-oncogene (MET, HGFR, Gene ID: 4233) following BRAF inhibition will reactivate mitogenic and survival pathways [52, 53, 127, 128]. A frequent observation in BRAF inhibitor resistance is coincidence with G protein-, RAS-, or PI3K-mediated reactivation of the MAPK pathway at the level of MAP2Ks or MAP3Ks. In addition to altered gene expression, other non-genomic mechanisms of regulation include promoter methylation, histone modifications, non-coding RNAs, posttranslational modification at the protein level, or metabolic feedback [99]. Importantly, non-genomic MAPK drivers have the ability to bypass central MAPK molecules and activate a similar program of MAPK effectors, without genomic activation of core or upstream factors such as BRAF in the MAPK pathways.

## Non-genomic MAPK subtype and oncogenic signaling in uveal melanoma

There is a striking similarity of the non-genomic MAPK subtype and oncogenic signaling in uveal melanoma. Uveal melanomas have less frequent somatic mutations and SCNAs in a much lower frequency than cutaneous melanoma. While BRAF and NRAS are usually of wild-type status [129], somatic mutations and SCNAs affecting G protein signaling are observed [130, 131]. However, the non-genomic MAPK program is heterogeneous and difficult to diagnose and treat, since similar mechanisms to MAPKi resistance might be utilized [132]. In uveal melanoma, combined inhibition of MAP2K1 and AKT results in synergistic anticancer activity of G protein-mutant tumors. In contrast, BRAF inhibition of the same tumors can enhance mitogen-activated protein kinase 1 (MAPK1, ERK, Gene ID: 5594) signaling, emphasizing the importance of personalized, genotype-based approaches. The effector program common to genomic MAPK and non-genomic MAPK subtypes is illustrated by activation of the RAC1 and AKT signaling axis, as well as activation of the E2F family transcription factors governing cell cycle progression. Total mutation burden as well as the fraction of genome altered are elevated with loss of tumor suppressors. Tumor suppressors exemplify the close connection between UV-related CPD-mediated nucleotide transversion with age-related accumulated genomic damage present in CSD melanoma. However, strict mutual exclusivity of major melanoma tumor suppressors NF1, PTEN, TP53, or CDKN2A to non-CSD melanomas is not observed, possibly due to parallel and/or redundant signaling. Therefore, loss of tumor suppressors does not suggest clinically actionable stratification or justify independent subtypes in this framework.

## Major signaling pathway alterations in melanoma

**Fig. 6:**
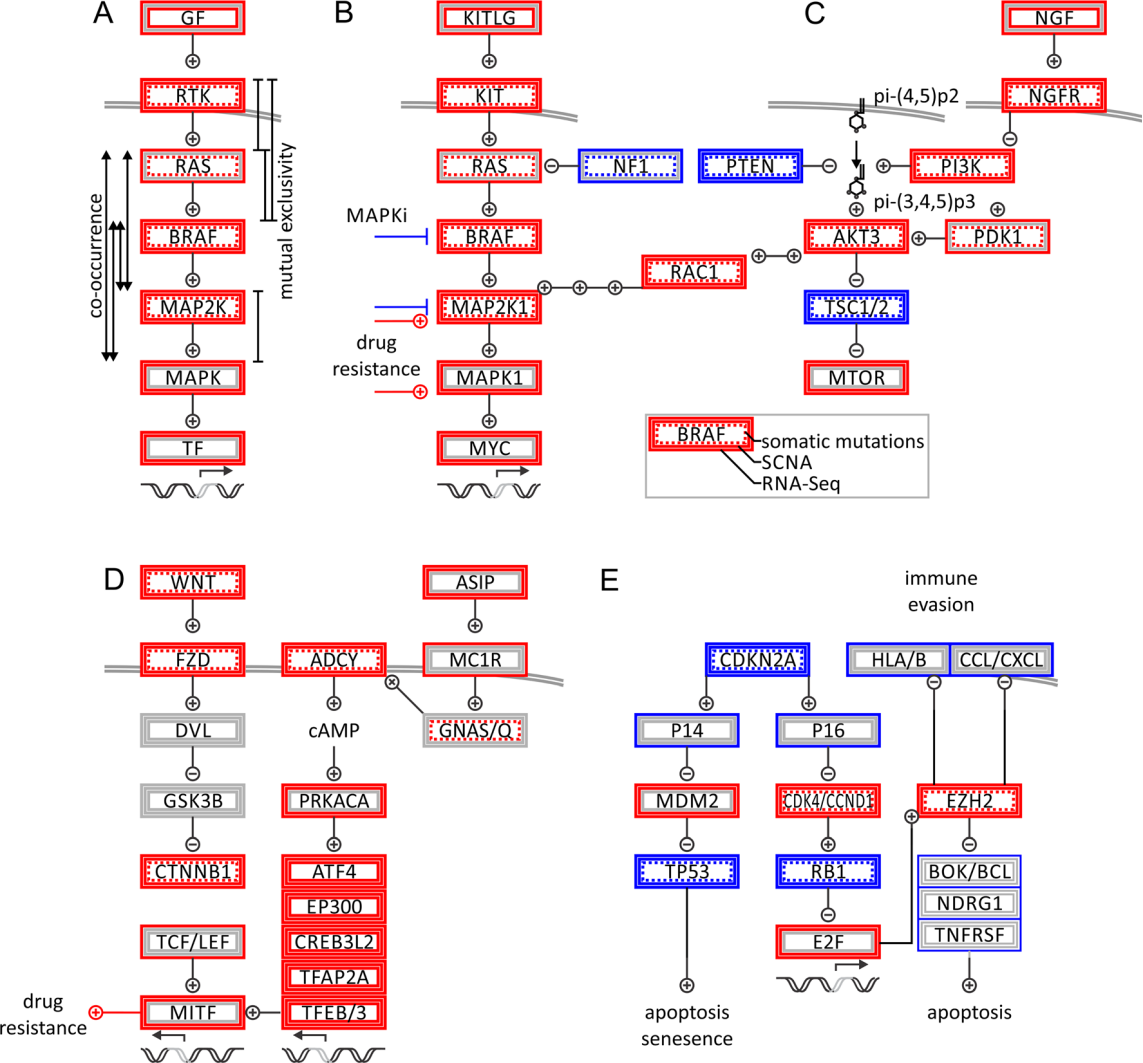
Pathway analysis of genomic alterations in malignant melanoma. **a** Oncogenic alterations of MAPK pathway is illustrated by somatic mutations, SCNAs, and transcriptional alterations (from *inner box* to *outer box*; see *legend*). Genomic activation and inactivation are shown in *red* and *blue*, respectively. Genomic co-occurrence and mutual exclusivity points to multiple parallel signals within pathway. **b** MAPK pathway. **c** PI3K pathway. **d** WNT and G protein signaling. **e** Cell cycle, senescence, and apoptotic signaling. Signaling maps of melanoma and melanogenesis highlight predominant oncogenes and tumor suppressors, in *red* and *blue*, respectively

**Fig. 7:**
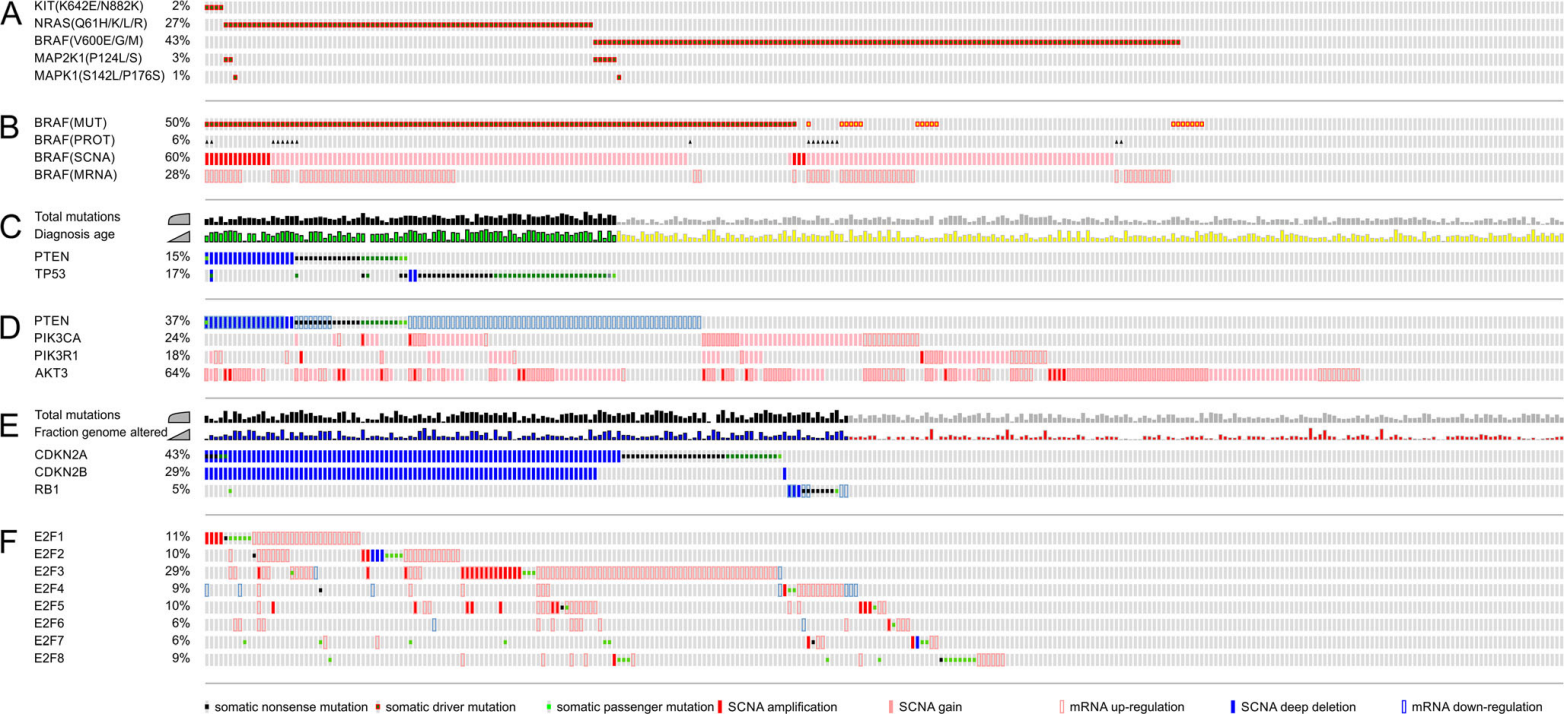
Molecular signatures and genomic alterations in malignant melanoma. Oncogenic alterations of **a** MAPK pathway; **b** BRAF at the mutational level (MUT), the protein level (PROT), the level of somatic copy number alterations, and at the gene expression level; **c** tumor suppressors PTEN and TP53 correlating with total mutation burden and age at diagnosis; **d** the PI3K/PTEN/AKT axis; **e** cell cycle inhibitors and tumor suppressors CDKN2A, CDKN2B, and RB1 correlating with total mutation burden and fraction of the genome altered; and **f** the E2F family of cell cycle-related transcription factors. Somatic mutations are marked as *dots*. SCNAs are highlighted as *red* and *blue bars* for amplification and loss, respectively. Significant deviation of mRNA gene expression by more than two standard deviations is marked with *transparent box* for each skin cutaneous melanoma (SKCM) patient of The Cancer Genome Atlas (TCGA) cohort

By taking all available data levels from genomics to proteomics into consideration, it is possible to prioritize signaling pathways that are consistently altered in malignant melanoma (Fig. 6). The MAPK kinase pathway presents itself as recurring theme. Intriguingly, driver mutations are not organized in a strict mutually exclusive fashion (Fig. 6a). While signals are processed by subsequently operated NRAS and BRAF switches which are mutually exclusive, there are alternative modes of activation and therefore co-occurrence of driver events in the subsequent kinase cascade. Such alternative modes coincide with re-wiring of pathways observed in melanoma drug resistance, where small molecules fail to completely block signaling, despite upstream kinases being dead (Fig. 6a, b). The picture is further complicated by interconnected signals of MAPK, RAC, AKT, PI3K, and NGFR pathways (Fig. 6b, c). RAC1 has been recognized as a frequently mutated melanoma gene by next-generation sequencing studies [21]. RAC1 mutations are present in all melanoma subtypes, which can be explained by its position at the crossroads of KIT/NRAS/BRAF/MAPK signaling and the PI3K/PTEN/AKT axis (Fig. 7). Therefore, RAC1 activation is independent of BRAF blockage and is able to induce mitogenic events.

## Signaling maps of melanoma and melanogenesis highlight predominant oncogenes and tumor suppressors

Melanocytes are derived from a neuroectodermal lineage providing them with a unique gene-expression signature and making them distinct from epithelial or non-epithelial cancers. Part of the melanocyte program is migration from the neural crest into ectoderm, differentiation, and clonal propagation of pigment-producing cells. Melanoma cells express neuroectodermal marker transmembrane receptors such as nerve growth factor receptor (NGFR, CD271, neurotrophin receptor, Gene ID: 4804), which has been found to be a marker for melanoma-initiating cells [133] (Fig. 6c). Melanogenesis and melanoma progression are interconnected by activation of wingless (WNT), melanocortin 1 receptor (MC1R, Gene ID: 4157), and melanogenesis-associated transcription factor (MITF, Gene ID: 4286) signaling. Not surprisingly, genomic profiles of melanoma and melanocytes overlap in transcriptional drivers of cellular mobility and proliferation, responsible for invasiveness and homing of melanoma [134–138] (Fig. 6d). Deregulation of melanogenesis can either contribute to dedifferentiation with loss of pigmentation comparable to a mesenchymal-epithelial transition, or elevated pigmentation and mitogenesis. Tyrosinase is essential for pigment synthesis in melanocytes and is an established antigen in melanoma. The roles of pigmentation in melanoma can be paradoxical: elevated tyrosinase expression can induce an auto-immune response leading to metastatic regression. Once tyrosinase-negative cells are able to escape this immune recognition, relapse and disease progression occur. Tumor suppressors are important safeguards of cells. The CDKN2A gene locus is frequently affected by copy number loss, non-sense mutations, and splicing aberration. Its two gene products, P14ARF and P16INK4A, control TP53 and RB transcriptional corepressor 1 (RB1, retinoblastoma protein 1, Gene ID: 5925) mediating cell cycle signaling [139, 140], respectively. Various genomic and non-genomic changes can lead to a deregulated cell cycle clock and apoptotic signaling (Figs. 6e and 7a–e). Inactivating mutations or promoter methylation of tumor suppressor RB1 and cell cycle inhibitors overwrite cell cycle checkpoints. Amplification and transcriptional upregulation of cyclins and cyclin-dependent kinases lead to RB1 phosphorylation and cell cycle progression. The tumor suppressor and pocket protein RB1 usually restricts the cell cycle progression by protecting and inhibiting the oncogenic E2F transcription factor family. Similarly, amplification and transcriptional upregulation of E2F transcription factors promote the cell cycle (Fig. 7f). Captivatingly, E2Fs are engaged in feedback loops by controlling cell cycle, anti-apoptotic survival pathways, and epigenetic regulators. E2F transcription factors including their main representative E2F1 are upstream regulators of the epigenetic polycomb complex around EZH2 constituting an important link to maintenance of the stem cell-like state of melanoma cells [141]. In addition, EZH2 has the ability to interfere with apoptotic pathways and immune evasion by repressing tumor suppressors, immune, cytokine and chemokine receptors (Fig. 6e). Signaling maps in combination with precision medicine profiles of cancer patients provide rational insights in treatment options and highlight predominant hubs of oncogenes and tumor suppressors (Figs. 3, 4, 5, 6, and 7).

## Conclusion

Melanoma precision medicine has significantly benefitted from large-scale sequencing efforts. Exome-wide and genome-wide sequencing initiated genomic biomarkers, targeted therapeutics, and encouraging clinical results. Collaborative, multiplatform genome sequencing approaches in cancer will be integrated into clinical practice in the near future. Molecular systems biology insights will impact prevention, diagnosis, and management of melanoma. Building a precision medicine framework is an investment in improved diagnostics and treatment protocols. Advanced molecular, high-throughput platforms fuel systems biology interpretation and enable novel genotype-phenotype associations in human melanoma. Cancer precision medicine still has to overcome challenges to promote the effective integration of genomic technology into clinical oncology practice. Melanoma is either characterized by frequent genomic alterations or genotypic heterogeneity. Therefore, acquired genomic precision data requires integration with cancer databases and clinical statistics. The objective is to distinguish between cancer drivers with clinically actionable targets or phenotypically neutral events, so that the treating precision medicine team can prioritize which clinical decisions should be pursued in order to provide cancer care that is personalized to the unique genome of the patient. Currently approved mono- and combination therapies are promising, and molecular insights from precision medicine have the ability to suggest orthogonal treatment targeting epigenetics and metabolism to overcome drug resistance and immune evasion.
